# Dissolution of Continuity of Ethical Governance in Data Science Health Research: Diagnostic and Response Framework for Lifecycle Oversight

**DOI:** 10.2196/99980

**Published:** 2026-09-29

**Authors:** Clement Adebamowo, Sally N Adebamowo, Adeola Akintola, Peter Ikhane, Simisola Akintola, Temidayo Ogundiran, Ayodele Jegede, Olusegun Adeyemo, Shawneequa Callier, Muhammad K Imam-Tamim, Ibrahim Uthman, BridgELSI Project as part of DSI Africa Consortium

**Affiliations:** 1 Department of Epidemiology and Public Health University of Maryland School of Medicine Baltimore, MD United States; 2 Greenebaum Comprehensive Cancer Center University of Maryland School of Medicine Baltimore, MD United States; 3 Department of Research Center for Bioethics and Research Ibadan Nigeria; 4 Department of Bioethics and Medical Humanities Faculty of Multidisciplinary Studies University of Ibadan Ibadan, Oyo State Nigeria; 5 Department of Private and Property Law Faculty of Law University of Ibadan Ibadan, Oyo State Nigeria; 6 Department of Surgery Faculty of Clinical Sciences University of Ibadan Ibadan, Oyo State Nigeria; 7 Department of Sociology Faculty of Social Sciences University of Ibadan Ibadan, Oyo State Nigeria; 8 Department of Clinical Research and Leadership School of Medicine and Health Sciences George Washington University Washington, DC United States; 9 Department of Islamic Law Faculty of Law University of Ilorin Ilorin, Kwara State Nigeria; 10 Department of Arabic and Islamic Studies Faculty of Islamic Studies University of Ibadan Ibadan, Oyo State Nigeria; 11 See Acknowledgments

**Keywords:** continuity dissolution, data science health research, lifecycle oversight, representational veracity, continuity trap, artificial intelligence governance, AI governance, health data ethics

## Abstract

Traditional research ethics governance was designed for bounded protocols, identifiable investigators, and temporally limited encounters with human participants. Data science health research (DSHR) disrupts that architecture because health data, biological materials, computational representations, and models persist, travel, combine, and acquire new uses across time. In this viewpoint, we use DSHR broadly to include research using large health datasets and adjacent secondary uses of health data, models, and biological materials, including learning health systems, public health surveillance, quality improvement, operations, commercial product development, and artificial intelligence (AI)–enabled translational uses that rely on health data or material lineages. We introduce ethical governance continuity dissolution (EGCD), the progressive and sometimes irreversible loss of domain-specific governance authority across a data, model, or biological material lineage, such that no coherent set of actors, instruments, or community processes can authorize, constrain, monitor, adjudicate, or remediate current use in relation to the persons and communities of origin. EGCD is distinct from consent staleness, function creep, contextual integrity violation, algorithmic drift, or a single defective data use agreement. It is the systemic condition in which several such failures together compromise the authority needed to govern current use. Building on our prior work on representational veracity and the continuity trap, we propose a diagnostic architecture that assesses six governance authority domains, applies three diagnostic criteria, scores each domain from 0 to 3 within a Continuity Authority Matrix (CAM), and stages severity from 0 to 4. We also propose an Ethical Continuity Governance and Response Mechanism (ECGRM) comprising a continuity registry, the CAM, trigger-based review, a Data Lifecycle Governance Officer function, a Continuity Dissolution Review Board, corrective and preventive action, cross-institutional audit, and community-governance integration. We use a publicly reported Royal Free–DeepMind Streams example to show how domain scores can identify stage 2 or 3 risk without converting the framework into a retrospective legal judgment. Implementation is proportionate, so these functions may operate within existing structures, particularly in underresourced institutions. The score and staging thresholds are conceptual triage aids, not validated metrics or automated determinations of ethical permissibility, and they require empirical validation and interrater reliability testing. Their purpose is to prevent the silent loss of governance authority while data, models, and biological materials continue to affect the lives, identities, and community standing of the persons and groups from whom they were derived. Lawful public health surveillance under a competent authority is a legitimate governance handoff, not EGCD; EGCD arises when authority is not transferred, traceable, accountable, or remediable. This viewpoint is addressed to research ethics committees and institutional review boards, data access committees, data stewards, biobank and registry leaders, AI governance teams, regulators, funders, community governance bodies, and investigators.

## Introduction

Data science health research (DSHR) uses statistical, computational, machine learning, and artificial intelligence (AI) methods to generate knowledge from heterogeneous health-related data. These data include electronic health records, cohort data, registries, claims, genomics, biospecimens, imaging, social media traces, mobile device and wearable sensor data, administrative records, and biological materials transformed into derivative resources, such as induced pluripotent stem cell lines. In this viewpoint, we used DSHR broadly to include research using large health datasets and adjacent secondary uses of health data, models, and biological materials when those activities rely on health data or material lineages. The term “DSHR” therefore includes research, development, quality improvement, operations, learning health systems, public health surveillance, and commercial product development when these activities generate ethical questions about secondary use, transfer, representation, or downstream deployment [1-3].

Traditional research ethics governance was designed for bounded studies. A protocol is written, risks and benefits are assessed, consent is obtained or waived, recruitment proceeds, data are collected, and a study eventually closes. Institutional review boards (IRBs), research ethics committees (RECs), data access committees (DACs), and related bodies were built around this protocol-centered model [4,5]. DSHR does not conform to this paradigm. Data collected in one context are reanalyzed decades later, linked to other sources, transferred across institutions, used to train models, converted into biological derivatives, or deployed in clinical and commercial settings. This produces a structural mismatch between a point-in-time governance architecture and a longitudinal data-model-material lifecycle [4-7].

Many elements of this mismatch have been examined, but the literature has not converged on a single diagnosis, and the debate remains active. Systemic oversight has been proposed because ethical challenges arise throughout the data-processing lifecycle rather than only at initial review [7]. Big data ethics scholarship shows that biomedical uses of large datasets raise a wide and contested range of concerns spanning privacy, ownership, control, epistemic quality, justice, commercial power, and collective consequences, and no single account commands consensus [8]. RECs face persistent oversight and functional gaps when health data are de-identified, aggregated, linked, or reused outside the original protocol [6]. Guidance on the ethics of AI often converges on high-level principles, yet those principles diverge in interpretation, institutional allocation, and enforcement, and they struggle to anticipate the still-unfolding risks and benefits of rapidly changing technologies [9,10]. Debates about informed consent remain equally unresolved. Broad consent, dynamic consent, and meta-consent offer different ways to govern secondary use, but each carries burdens, exclusions, and implementation limits [11-14]. Some scholars doubt that informed consent can retain its protective role in big data and AI environments, where future uses, inferences, and affected groups cannot be specified at collection [15,16], while others defend consent for health record research as an expression of respect, control, and accountability, even when uses are low risk or socially valuable [17]. We treat consent authorization as one governance authority among several rather than the sole ethical anchor.

We argue that DSHR ethics requires a concept for the systemic condition that emerges when governance authorities weaken and interact across the lifecycle. We call that condition ethical governance continuity dissolution (EGCD). Continuity dissolution is not simply a stale consent form, an unreviewed dataset, a drifting algorithm, a contextual integrity violation, or a deficient data access agreement. It is the condition in which *the authorities that make governance possible lose their capacity to authorize, constrain, monitor, adjudicate, or remediate a data, model, or biological material lineage across its lifecycle*. When continuity dissolution occurs, no single governance actor or instrument can account for the ethical relationship between the derivative object and the persons and communities from whom it originates.

Our argument builds on two prior concepts from our program of work. *Representational veracity* refers to the extent to which banked data, including identifiers, clinical variables, models derived from them, and population descriptors, continue to accurately and responsibly represent the biological, social, and value-based characteristics of the persons and phenomena they describe, at the point of use and not merely at the point of collection [18]. The *continuity trap* is a review-stage governance error in which a salient signal of continuity in one domain, often a stable identifier, provenance trail, approval number, or data use agreement, is treated as sufficient evidence of ethical continuity overall, causing inquiry into other domains to close prematurely [19]. Continuity dissolution is broader than either concept because it names the progressive loss of governance authority that can follow when multiple domains become disconnected from data origin, current use, affected communities, and practical remedy.

This viewpoint aims to provide a diagnostic and response framework for recognizing, staging, and managing continuity dissolution in DSHR and adjacent secondary uses of health data, models, and biological materials. We first define continuity dissolution and differentiate it from adjacent concepts. We then assess six governance authority domains, link them to three diagnostic criteria, integrate them into a scored Continuity Authority Matrix (CAM) and a five-level staging system, and specify an Ethical Continuity Governance and Response Mechanism (ECGRM) that institutions can adapt across regulatory, infrastructural, and resource settings. Our intended audience includes IRBs and RECs, DACs, data stewards, biobank and registry leaders, health system data offices, AI governance teams, regulators, funders, community governance bodies, and investigators conducting DSHR worldwide.

### Definition and Conceptual Boundaries

#### Ethical Governance Continuity Dissolution

EGCD is the progressive and sometimes irreversible loss of domain-specific governance authority across a data, model, or biological material lineage, such that no coherent set of actors, instruments, and community processes can authorize, constrain, monitor, adjudicate, or remediate current use in relation to the persons and communities of origin. The definition identifies four characteristics of EGCD: (1) EGCD is progressive, that is, it accumulates through many ordinary lifecycle transitions rather than through a single breach; (2) it is lineage based, that is, the affected object may be a dataset, biospecimen, induced pluripotent stem cell line, trained model, model output, digital twin, synthetic dataset, federated model, or downstream clinical product; (3) it concerns the loss of governance authority rather than the loss of documentation, that is, a consent form, approval letter, data transfer agreement, model card, or provenance trail may remain intact, while the authority it once carried has eroded; and (4) it is relational, that is,: the operative ethical question is whether current use remains connected to the persons, groups, communities, and institutions from which the data or material originated [5,17,19].

A *governance authority* is the legitimacy-bearing capacity of an actor, instrument, rule, or community process to authorize, constrain, monitor, adjudicate, or remediate a use because it remains connected to the origin, current use, affected persons and communities, and practical remedial control. Governance authority may be legal, institutional, professional, community based, contractual, technical, or remedial. A *governance instrument* is the concrete artifact or mechanism through which such authority is exercised or documented. Governance instruments include consent forms, broad-consent language, waivers, IRB and REC decisions, DAC approvals, data use agreements, material transfer agreements, privacy notices, data protection impact assessments, fundamental rights or algorithmic impact assessments, model cards, community agreements, audit logs, monitoring protocols, withdrawal procedures, benefit-sharing agreements, and corrective and preventive action plans. An instrument can persist after the authority it once carried has become fragile, compromised, or dissolved [6,20].

EGCD occurs when several governance authorities fail together or when a critical authority fails and no remedial authority can restore governance. We deliberately avoid reducing ethical governance to either consent or regulatory compliance. Consent, legal basis, review jurisdiction, classification, representation, technical assurance, and remedy all matter, because a lineage may remain lawful in one respect while losing ethical continuity in another [6,7].

#### What EGCD Is Not and the Difference Between Handoff and Dissolution

Not every weakness of ethical governance amounts to EGCD. A stale consent form is not EGCD when active oversight, provenance, community engagement, and remedial authority remain functional. Algorithmic drift is not EGCD when the model is monitored, update procedures are authorized, deployment can be paused, and affected groups can be protected. A research or nonresearch classification dispute is not EGCD when a credible escalation pathway exists. EGCD is a higher-order condition—the failure of the governance assemblage rather than the failure of any single tool.

EGCD should not be inferred from the absence of individual consent alone. Public health surveillance, emergency response, legally authorized registry functions, quality improvement, and learning health system activities may be ethically justified under governance frameworks that differ from traditional research ethics [21-24]. A legitimate governance handoff occurs when a lineage moves from one ethically authorized governance framework to another, as when clinical or research governance transfers to a competent public health authority operating under a lawful surveillance mandate. The World Health Organization recognizes that public health surveillance can be ethically justified with identifiable data and without individual consent when valid and complete data are necessary, safeguards are in place, and surveillance operates under legitimate authority [23,24]. Such a handoff preserves ethical governance continuity, even when the consent model changes. EGCD is plausible only when the handoff fails, that is, when no receiving authority is identifiable, the legal or institutional mandate is unclear, classification is manipulated to avoid review, affected communities are unrepresented, technical assurance is absent, or no actor holds practical remedial control (Table 1).

**Table 1:** Comparison of EGCD^a^ with adjacent concepts and legitimate governance handoff.

Adjacent concept	Primary focus	How EGCD differs
Continuity trap	A specific diagnostic error: Visible continuity in one domain masks discontinuity elsewhere.	EGCD is the systemic condition that may follow multiple uncorrected traps and other lifecycle failures.
Representational veracity failure	Loss of epistemic or ethical fidelity between data or model and the persons or communities represented.	Representational failure is one domain of EGCD; EGCD adds authorization, oversight, classification, algorithmic assurance, and remedy.
Function creep	Expansion of a technology or dataset beyond its original purpose.	EGCD includes function creep only when expanded use also dissolves authority to authorize, monitor, or remedy.
Consent staleness or expiry	Original permission no longer maps to current use or values.	Authorization decay is one mechanism; EGCD requires broader authority failure or loss of remedial control.
Contextual integrity violation	Information flows violate context-specific norms.	EGCD describes the state in which the original context and its governing norms are no longer adequate anchors for the lineage.
Algorithmic drift or fairness drift	Performance or fairness changes after deployment.	EGCD includes drift only when no authority can monitor, update, pause, explain, or remediate it.
Systemic oversight	A lifecycle approach to oversight.	Systemic oversight is a remedy architecture; EGCD is the pathology that such oversight should diagnose and prevent.
Legitimate governance handoff	Authorized transfer between two lawful governance frameworks (eg, clinical care to public health surveillance).	A successful handoff preserves continuity; EGCD arises only when the receiving authority, mandate, representation, assurance, or remedy is absent.

^a^EGCD: ethical governance continuity dissolution.

### Diagnostic Architecture, the Continuity Authority Matrix, and Staging

#### Diagnostic Architecture

The EGCD framework is most useful when it prevents institutions from treating serious lifecycle governance failures as routine administrative irregularities. Its architecture is sequential and integrated. A lifecycle transition triggers review. Reviewers then assess the six governance authority domains within a single CAM, in which each domain is scored from 0 to 3 and supported by documentary evidence. The three diagnostic criteria determine whether EGCD is present, the summed domain scores and staging grade its severity, and the stage determines a proportionate ECGRM response (Figure 1).

Triggering transitions include secondary use outside the original consent or authorization context; linkage across datasets; cross-border transfer; transfer from academic to commercial actors; derivation into induced pluripotent stem cells, organoids, synthetic data, or model parameters; federated or privacy-preserving aggregation; deployment into clinical or public health operations; material change in model purpose; and end-of-lifecycle transfer, archiving, or retirement. A lineage should be evaluated for EGCD after any substantial lifecycle transition. Evaluation is especially warranted when existing governance records cannot readily demonstrate that current use remains authorized, reviewable, representationally valid, monitorable, and remediable. This is consistent with the broader shift from initial approval toward lifecycle and systemic oversight in big data health research and AI-enabled health technologies [6,7,25-27].

EGCD is present when all three of the following criteria are met:

- Governance-authority failure. Either at least two governance authority domains are compromised, or one critical domain is dissolved, and no effective remedial authority remains.
- External consequence. The current or proposed use has reasonably foreseeable consequences for identifiable persons, groups, communities, institutions, or health systems, beyond purely internal technical processing.
- Irreparability by existing instruments. Existing governance instruments cannot reconstitute an ethically meaningful relationship between the lineage and the persons or communities from whom it originated.

**Figure 1:**
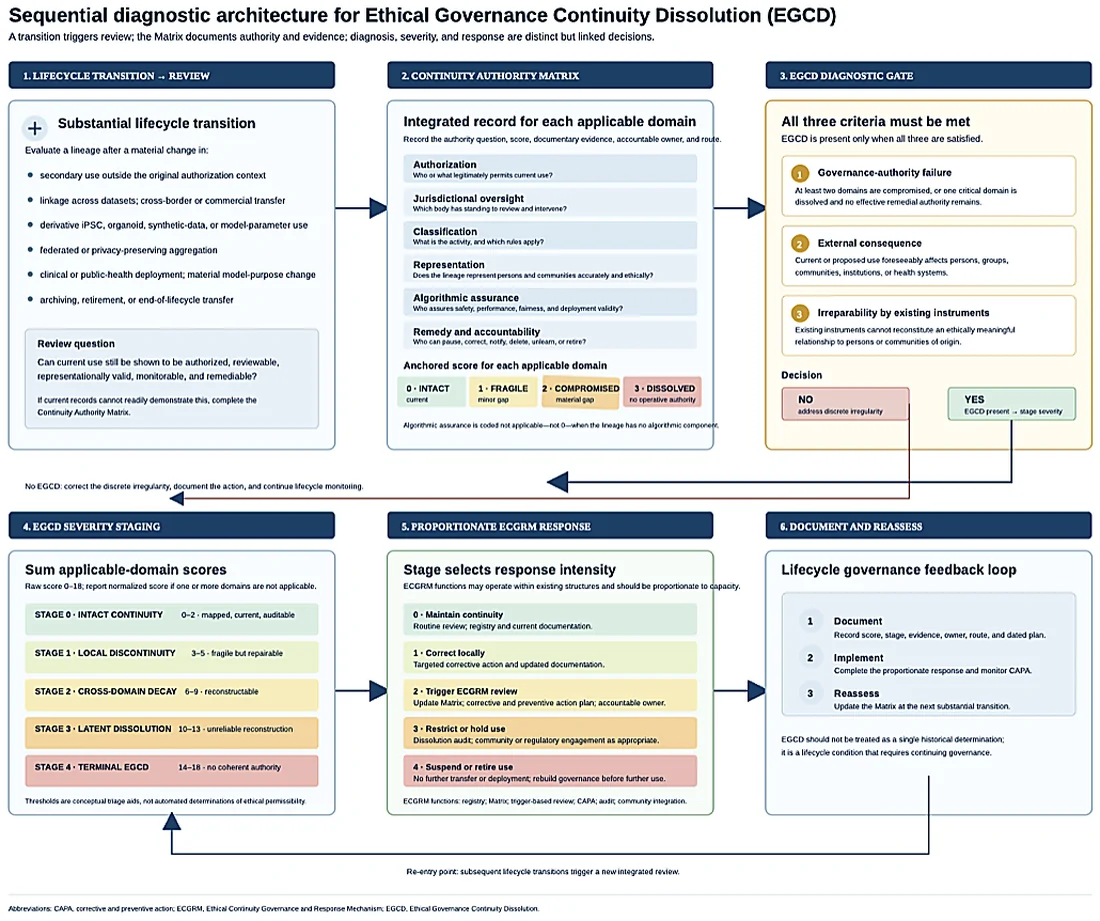
Sequential diagnostic architecture for EGCD. A substantial life cycle transition triggers review through the CAM. The three diagnostic criteria determine whether EGCD is present; applicable domain scores stage severity and select a proportionate ECGRM response. Decisions are documented and reassessed at subsequent life cycle transitions. CAM: Continuity Authority Matrix; EGCD: ethical governance continuity dissolution; ECGRM: Ethical Continuity Governance and Response Mechanism.

#### The Continuity Authority Matrix

The CAM is the integrated diagnostic and scoring instrument at the center of the framework. It combines the governance authority question for each domain, the common mechanisms and indicators of dissolution, the anchored 0-3 score, and the documentary evidence recorded for that score (Table 2). Each domain is scored 0 for intact, 1 for fragile, 2 for compromised, and 3 for dissolved. A domain that does not apply, such as algorithmic assurance in a purely biological lineage with no computational model or automated inference, is coded “not applicable” rather than scored 0. The matrix is completed for major secondary uses, data linkages, model training, cross-border transfers, commercial partnerships, federated learning projects, and derivative biological material uses, and it can incorporate an existing data protection impact assessment, fundamental rights impact assessment, IRB or REC decision, DAC decision, model card, data use agreement, or community agreement as evidence when those instruments remain current and applicable.

**Table 2:** CAM^a^: six governance authority domains, dissolution indicators, anchored scores, and evidence recorded.

Governance authority domain	Authority question	Common dissolution mechanisms and indicators	Anchored score (0-3)	Evidence recorded
Authorization	Who or what legitimately permits current use?	Consent expiry, scope drift, unanticipated AI^b^ use, commercial transfer, decay of community permission or social license to operate, waiver overuse.	0: current authorization covering use 1: minor scope distance 2: authorization not clearly covering use 3: no operative authorization and no reauthorization pathway	Consent, legal, or community basis; scope; reauthorization pathway
Jurisdictional oversight	Which body has standing to review and intervene?	IRB^c^ or REC^d^ exclusion because data are de-identified; DAC^e^ limited to access only; product development outside research review; cross-border gaps	0: accountable body with current jurisdiction 1: review overdue 2: no clear reviewing body 3: no body can review current use	Reviewing body, jurisdiction, and review interval
Classification	What is the activity, and which rules apply?	Boundary blurring among research, quality improvement, care, operations, surveillance, model maintenance, and commercialization	0: stable, justified classification 1: classification under review 2: classification changed without review 3: classification manipulated to evade oversight	Current classification, justification, and escalation pathway
Representation	Does the lineage still represent persons and communities accurately and ethically?	Descriptor drift, ontology or nosology change, provenance attenuation, aggregation across communities, proxy categories, loss of community standing	0: provenance and descriptors intact 1: minor descriptor drift 2: attenuated attribution or unmapped community 3: unrecoverable source attribution or invalid transfer to new populations	Descriptors, provenance, affected communities, and transferability
Algorithmic assurance	Who assures ongoing safety, performance, fairness, and deployment validity?	Model drift, fairness drift, silent updating, distribution shift, deployment beyond validation context	0: monitoring and update governance current 1: monitoring incomplete 2: widening subgroup gaps or absent update governance 3: no assurance for a deployed system; not applicable if no algorithmic component	Validation, monitoring, update protocol, and deployment constraints
Remedy and accountability	Who can pause, correct, notify, compensate, delete, unlearn, or retire?	Transfer without enforceable clauses, federated aggregation, untraceable model parameters, commercial product lock-in	0: identified actor with enforceable remedy 1: remedy partially specified 2: remedy unclear or unenforceable 3: no actor can suspend use or honor withdrawal	Remedial actor, enforceable powers, and corrective and preventive action owner

^a^CAM: Continuity Authority Matrix.

^b^AI: artificial intelligence.

^c^IRB: institutional review board.

^d^REC: research ethics committee.

^e^DAC: data access committee.

For each lineage, the matrix also records a lineage identifier, the lifecycle transition under review, the summed applicable domain score, the assigned stage, and a dated management plan with an accountable owner and escalation route. The matrix is thus at once the domain taxonomy, the scoring rubric, and the record of evidence and remedy.

#### Scoring, Staging, and Reliability

The summed score across applicable domains supports triage. When a domain is not applicable, institutions report both the raw applicable domain score and a normalized score, calculated as the observed score divided by the maximum-possible applicable domain score and multiplied by 18. Scores map to stages as follows: 0-2, intact continuity or low concern; 3-5, watch status; 6-9, continuity decay; 10-13, latent dissolution; and 14-18, terminal EGCD. These thresholds are sentinel triggers for deliberation, not automated determinations of ethical permissibility. The staging system in Table 3 grades severity and specifies a proportionate response for each stage.

**Table 3:** Staging system for EGCD^a^.

Stage	Label	Diagnostic description	Required response
0	Intact continuity	All governance authorities are mapped, current, auditable, and connected to practical remedial control.	Routine review; maintain registry and documentation.
1	Local discontinuity	One governance authority is fragile or outdated, but others can investigate and repair it.	Targeted corrective action and updated documentation.
2	Cross-domain decay	Two or more governance authorities are weakened after a lifecycle transition, but governance remains reconstructable.	Triggered ECGRM^b^ review, CAM^c^ update, and corrective and preventive action plan.
3	Latent dissolution	Use continues while authorization, oversight, representation, assurance, or remedy cannot be reliably reconstructed.	Restricted access or use hold, dissolution audit, and community or regulatory engagement, as appropriate.
4	Terminal EGCD	No coherent set of governance authorities can authorize, monitor, adjudicate, or remediate current use.	Suspend or retire use; prohibit further transfer or deployment; rebuild governance before any further use.

^a^EGCD: ethical governance continuity dissolution.

^b^ECGRM: Ethical Continuity Governance and Response Mechanism.

^c^CAM: Continuity Authority Matrix.

The boundary between stages 2 and 3 is an evidentiary threshold rather than a matter of intuition. A lineage is reconstructable when the responsible actors can identify the operative authority, locate the governing instruments, confirm the current classification, identify affected persons or communities with sufficient fidelity, determine whether technical or algorithmic assurance remains valid, and name an actor with enforceable remedial control. The Data Lifecycle Governance Officer function, described later, can make the initial triage recommendation, but a Continuity Dissolution Review Board or equivalent cross-functional body should adjudicate stage 3 and 4 determinations on a documented and reviewable basis. Because domain scores rely on judgment, institutions should use the anchored descriptors in Table 2, require documentary evidence for each score, conduct independent dual scoring for higher-risk lineages, and adjudicate discrepant scores through the Officer and Review Board pathway. Empirical validation should test interrater reliability using statistics appropriate to ordinal or categorical ratings, such as weighted Cohen κ, Fleiss κ, Gwet AC1 or AC2, or intraclass correlation coefficients, depending on the design and number of raters [28,29].

### Legal, Policy, and Ethical Framework Pluralism

Governance authority in EGCD is not limited to legal authority, but law and policy are important sources of continuity. National research regulations, such as the Common Rule, establish IRB and REC jurisdiction for human subject research [4,5]. Data protection law can require a lawful basis, transparency, rights management, and data protection impact assessments for high-risk processing; the General Data Protection Regulation (GDPR) creates the Data Protection Officer role for organizations whose core activities involve large-scale processing of special-category data or systematic monitoring [20]. The European Health Data Space establishes a framework for primary and secondary use of electronic health data, including health data access bodies and safeguards for reuse [30]. The European Union Artificial Intelligence Act adds obligations for high-risk systems, including fundamental rights impact assessment for specified deployers and post-market monitoring [31]. United States Food and Drug Administration guidance on AI-enabled device software functions and predetermined change control plans similarly reflects lifecycle governance, monitoring, and change management expectations [25,27].

These laws and frameworks are relevant, but they are not the only sources of governance authority. EGCD can arise where formal law is silent but institutional policy, community agreement, contractual authority, technical governance, or remedial control has dissolved. Legal compliance in one domain also does not establish ethical continuity across the lineage. The CAM indexes these distinct sources of authority and identifies when an existing legal or policy instrument can serve as evidence rather than be duplicated.

EGCD is compatible with pluralism among ethical frameworks. Lee [32] argues that public-health ethics can bridge traditional bioethics and environmental ethics, allowing different frameworks to become more salient depending on whether risks and benefits are individual, group based, ecological, or systemic. This is directly relevant to DSHR. Traditional bioethics may dominate when a lineage creates individualized risk or requires consent, public health ethics may be central when surveillance or population-level benefit is at stake, and environmental or systems ethics may become relevant when AI infrastructures, energy use, platform power, or nonhuman impacts matter. EGCD does not replace these frameworks. It is a continuity diagnostic that asks which governance authority has dissolved, and which normative framework must be re-engaged to restore it, because authorization, oversight, classification, representation, assurance, and remedy may implicate different persons, publics, institutions, and systems at different points in time.

### Illustrative Domains and a Worked Example

The following settings illustrate where EGCD recurs: multisource linked administrative health data; commercial AI training on clinical data; genomic consortia, biobanks, and derivative biological materials; federated learning and privacy-preserving analytics; and AI-inferred identities and digital twins. The response options are drawn from the six governance authority domains and are intended to repair specific authority deficits rather than impose a single governance template. Unless anchored in law or formal guidance, they are presented as possible corrective responses rather than universal requirements.

#### Multisource Linked Administrative Health Data

Linked administrative health platforms combine clinical records, claims, registries, pharmacy data, mortality records, social care data, and sometimes genomic or geospatial data. These systems create public value, but they can also produce EGCD. Authorization weakens when clinical care data become research, audit, operations, policy, or commercial development assets. Oversight weakens because no single body may review the linked platform as a whole. Classification becomes unstable because the same use may be described as care improvement, research, public health surveillance, product development, or service planning. The challenge is not the existence of linked data but the absence of a lifecycle mechanism that maintains authority across linked uses [30,33]. Response options include establishing a continuity registry for the platform, completing a CAM for each major use category, publishing secondary-use pathways, convening periodic cross-functional review, and constituting community advisory mechanisms proportionate to platform scale (authorization, oversight, classification, and remedy).

#### Commercial AI Training on Clinical Data

Commercial model training intensifies EGCD because it creates strong incentives to treat clinical data as a product development resource rather than a relational health record. Authorization may not cover commercial model development. Oversight may be bypassed when the activity is classified as product development rather than research. Algorithmic assurance may be fragile when models are updated, redeployed, or embedded in proprietary systems. Remedy may be weak when contracts do not specify audit rights, withdrawal conditions, deletion, model update governance, or downstream deployment limits [3,26,27,34]. Response options include specifying permitted and prohibited uses, requiring update governance, reporting subgroup performance, enabling feasible withdrawal or deletion, mandating postdeployment monitoring and audit rights, publishing transparency information, and naming an actor empowered to pause or terminate use (authorization, oversight, classification, algorithmic assurance, and remedy).

#### Genomic Consortia, Biobanks, and Derivative Biological Materials

Genomic consortia and biobanks are paradigmatic EGCD settings because they combine long time horizons, broad consent, technological change, population-level inference, cross-border sharing, and derivative materials. A biospecimen collected for one form of research may become a renewable induced pluripotent stem cell line, an organoid, a genome sequence, polygenic-risk-score input, or a model training sample. The original consent may be legally broad yet ethically thin once the object, use, community consequences, and commercial context change. Representation is vulnerable because aggregate signals may be detached from the communities whose data made them possible, while downstream tools may be deployed in populations for which they were not validated [11,13,35-38]. Response options include reviewing consent scope, auditing representational veracity, analyzing community standing, registering derivative materials, conducting cross-institutional dissolution audits, and specifying benefit-sharing and remedy provisions (authorization, representation, oversight, and remedy).

#### Federated Learning and Privacy-Preserving Analytics

Federated learning, differential privacy, secure multiparty computation, and related methods can reduce privacy risks, but they do not eliminate ethical governance obligations. The claim that data never move can itself become a continuity trap when it leads institutions to overlook functional secondary use, model extraction, gradient contribution, downstream deployment, and group consequences. Authorization may be weak because data are repurposed in place. Representation may dissolve when local contributions are absorbed into a global model. Remedy may be unclear when a site withdraws after model aggregation [19,39,40]. Response options include treating federated participation as a lifecycle transition, documenting authorization mapping, governing at the protocol level, recording model contributions, governing updates, specifying exit clauses, and reviewing deployment context (authorization, representation, algorithmic assurance, and remedy).

#### AI-Inferred Identities and Digital Twins

Secondary use governance becomes more fragile when systems infer attributes that were never collected explicitly. A data subject may have consented to share typing patterns, imaging, clinical notes, or wearable measurements without authorizing inference of mental health status, cognitive decline, fertility risk, or future disease. Digital twins raise similar concerns because they create persistent simulations that may continue after consent changes, death, incapacity, or institutional transfer. EGCD arises when governance cannot determine whose identity is represented, which inferences are authorized, who can contest them, and whether the derivative object can be paused, deleted, corrected, or retired [2,3,41]. Response options include reviewing inference scope, restricting sensitive inferences, honoring future use data directives, requiring human oversight of automated consent tools, and adopting clear policies for withdrawal, digital retirement, and posthumous or incapacity-related governance (authorization, representation, algorithmic assurance, and remedy).

#### Worked Example: Royal Free–DeepMind Streams

The Royal Free–DeepMind Streams case shows how the CAM can be used prospectively as a diagnostic rather than a retrospective condemnation. Publicly reported accounts state that the Royal Free London National Health Service (NHS) Foundation Trust entered a data-sharing arrangement with DeepMind for the Streams acute kidney injury application and that large volumes of patient data were made available for that purpose. The Information Commissioner’s Office later found that the trust had not complied with data protection requirements, including the need for sufficient patient information about how data were used; contemporaneous commentary described contested classification, transparency, and legal basis issues [42-44]. Applied prospectively, the matrix asks whether the transition from clinical care records to digital product development and clinical deployment preserved the six governance authorities. Scoring the publicly reported facts against the anchored descriptors yields a provisional applicable domain score consistent with stage 3 triage if use continues without reconstruction (Table 4). Effective corrective action could return the lineage to stage 2 or lower.

**Table 4:** Illustrative application of the CAM^a^ to the Royal Free–DeepMind Streams case.

Governance authority domain	Illustrative findings from public records	Provisional score	Response option
Authorization	Patient-facing transparency and legal basis were contested.	2	Reconstruct legal and community basis; update notices; and document permitted uses.
Jurisdictional oversight	Governance responsibility sat across care delivery, data protection, product development, and deployment.	2	Trigger cross-functional review and define an accountable oversight body.
Classification	The activity could be characterized as care support, application development, audit, or innovation.	2	Record the classification and escalation pathway in the matrix.
Representation	Representation risk was lower than in genomic portability cases but still required population and clinical context mapping.	1	Document the affected population, clinical setting, and transfer limits.
Algorithmic assurance	Assurance depended on the system function; monitoring and update governance were not explicit.	1	Specify validation, monitoring, and update responsibilities.
Remedy and accountability	Remedy required enforceable pause, audit, transparency, and contract correction powers.	2	Assign a remedial actor and corrective action owner and audit completion.

^a^CAM: Continuity Authority Matrix.

The provisional score of 10 out of 18 is consistent with stage 3 triage if use continues without reconstruction. Note that this illustration rests on public records and is neither a full empirical case study nor a legal determination.

### Ethical Continuity Governance and Response Mechanism

The ECGRM is the institutional response to EGCD. It moves beyond point-in-time review without assuming that every participant must be recontacted for every future use. Its objective is lifecycle authority. Each material change in the use of data, a model, or a biological material should have an identifiable authority structure capable of authorization, oversight, classification, representation, assurance, and remedy [7,25,26]. The mechanism should be proportionate to lineage risk and institutional capacity, and its components can operate as functions within existing offices and committees rather than as new bureaucracy in every setting.

Every institution conducting DSHR should maintain a continuity registry of active datasets, models, derivative biological materials, data-sharing agreements, consent instruments, access approvals, deployment contexts, community governance mechanisms, and responsible stewards. In low-resource settings, the registry can be a structured spreadsheet or REDCap instrument; in high-capacity settings, it can integrate with data catalogs, machine learning operations systems, contract management systems, and IRB or DAC platforms. The registry is the minimum infrastructure needed to make lifecycle authority visible and auditable [26,45]. The CAM, specified in the *Diagnostic Architecture, the Continuity Authority Matrix, and Staging* section, is the diagnostic and scoring core of the ECGRM and draws on existing instruments as evidence rather than duplicating them. To make that boundary explicit, Table 5 distinguishes the matrix and the Data Lifecycle Governance Officer from adjacent legal and technical instruments and roles.

**Table 5:** CAMa and Data Lifecycle Governance Officer compared with adjacent instruments and roles.

Instrument or role	Primary object	Timing	Relation to the matrix and the officer
Data protection impact assessment	High-risk personal data processing	Before high-risk processing; updated when risk changes	Supplies privacy evidence for authorization, oversight, and remedy; does not by itself assess full lineage continuity
Fundamental rights or algorithmic impact assessment	Fundamental rights or algorithmic impacts of high-risk AI^b^	Before deployment and when use materially changes	Supplies evidence for algorithmic assurance, classification, representation, and remedy
IRB^c^ or REC^d^ review	Human subject research protocol	Initial review, continuing review, or amendment	Supplies oversight evidence when current use remains within review jurisdiction
DAC^e^ decision	Access to data or samples under repository rules	At access request and renewal	Supplies access governance evidence but may not govern downstream deployment or derivative uses
Model card	Model description, performance, limitations, and intended use	At release or substantial update	Supplies algorithmic assurance evidence; does not create authorization or remedy authority
Data use or material transfer agreement	Contractual permissions and obligations for transfer	At transfer and amendment	Supplies continuity clauses when it includes triggers, audit, community governance, benefit sharing, and remedy
Data Protection Officer	Data protection compliance and advisory function	Ongoing	Does not conflict with the officer; may host or contribute to the function where remit and capacity allow
Data Lifecycle Governance Officer	Lifecycle coordination across the six EGCD^f^ domains	Triggered and ongoing	Maintains the registry, coordinates the matrix, initiates review, tracks corrective action, and reports continuity risk

^a^CAM: Continuity Authority Matrix.

^b^AI: artificial intelligence.

^c^IRB: institutional review board.

^d^REC: research ethics committee.

^e^DAC: data access committee.

^f^EGCD: ethical governance continuity dissolution.

Institutions conducting DSHR should designate a Data Lifecycle Governance Officer function. The officer does not replace an IRB, REC, DAC, Data Protection Officer, privacy office, legal office, or community advisory body. Core duties include maintaining the registry, ensuring completion of the CAM, identifying trigger events, initiating EGCD review, coordinating corrective actions, and producing an annual continuity report. In most institutions, this function can be assigned to an existing research governance, data stewardship, or compliance office rather than being established as a separate post. When the matrix identifies stage 2 or higher risk, review should shift to a Continuity Dissolution Review Board or equivalent cross-functional process, including REC or IRB expertise, data access governance, privacy and legal expertise, AI or informatics expertise (where relevant), domain science, data stewardship, and community representation when community consequences are plausible. The board determines whether authority can be restored, whether use should be restricted, or whether terminal EGCD requires suspension or retirement [6,7].

Management of EGCD should be documented in a corrective and preventive action plan. Possible actions include reconsent, meta-consent, community re-engagement, new ethics review, use limitation, descriptor repair, ontology harmonization, provenance reconstruction, restoration of community standing, fairness and performance audit, model retraining, model quarantine, access suspension, deletion, machine unlearning (where feasible), transparency reporting, benefit-sharing review, and retirement of the lineage. These actions translate consent, oversight, fairness, and AI lifecycle governance into an institutional response pathway [12,13,27,46].

### Global Implementation

EGCD is a global phenomenon. High-income settings may have sophisticated regulation, but they also operate high-volume data infrastructures, commercial AI partnerships, and complex learning health systems that accelerate dissolution. Middle-income settings may experience data infrastructure growth that outpaces legal and ethics capacity. Low-resource settings may face external data extraction, limited postapproval monitoring, underresourced community governance, and weak negotiating power in international collaborations. The ECGRM should therefore scale by lineage risk and institutional capacity rather than by national income category [1,3,21,22,30,31]. A minimum viable mechanism can be deliberately lean, comprising a structured registry, a CAM for high-risk lineages only, a named coordinating function, a trigger checklist, and an annual review. Institutions should not create a standing review board when existing IRBs, RECs, DACs, data governance committees, or legal and compliance offices can perform the function under defined terms of reference. External collaborators should treat weak postapproval monitoring, absent community governance, or unenforceable remedy as design problems to be corrected rather than as reasons to bypass local authority (Table 6).

**Table 6:** Proportionate implementation of the ECGRM^a^.

Implementation tier	Required elements	Function or post?	Appropriate settings
Minimum viable	Continuity registry, CAM^b^ for high-risk uses, named officer-equivalent function, trigger checklist, annual review	Functions embedded in existing offices or committees	Any institution conducting DSHR^c^, including low-resource settings
Intermediate	Standing or convened review board, domain scoring, documented corrective and preventive action, community governance mapping, cross-institutional audit clauses	Existing committees with added continuity terms of reference	Research-intensive institutions, biobanks, genomic consortia, national registries
Advanced	Automated registry integration, machine learning operations monitoring, fairness dashboards, contract trigger alerts, independent audits, public transparency reports	Dedicated functions perhaps justified	Large health systems, national data platforms, AI^d^ development partnerships, high-volume repositories

^a^ECGRM: Ethical Continuity Governance and Response Mechanism.

^b^CAM: Continuity Authority Matrix.

^c^DSHR: data science health research.

^d^AI: artificial intelligence.

International collaborations should include continuity clauses in data transfer and consortium agreements, specifying authority mapping, trigger events, audit rights, community engagement, benefit sharing, and remedies for downstream use. Existing responsible data-sharing frameworks provide useful foundations, but EGCD requires an explicit continuity layer that tracks authority after data and models cross institutional boundaries [1,45].

## Discussion

### Summary

EGCD reframes familiar ethical problems as interacting components of a systemic lifecycle pathology. Consent staleness, function creep, review evasion, boundary blurring, representational attenuation, fairness drift, and remedy failure are not merely separate defects requiring separate fixes; in DSHR, they can reinforce one another. Broad consent may permit future research, but when the activity is reclassified as product development, no IRB retains jurisdiction, the community of origin is lost, the model drifts after deployment, no actor can pause downstream use, and the problem exceeds consent and becomes governance continuity dissolution across the lineage [6,7,41,46].

The relationship to systemic oversight is close. Proponents of systemic oversight argue that ethical review must be adaptive, flexible, monitored, responsive, reflexive, and inclusive across the data lifecycle. EGCD complements that view by naming the condition that systemic oversight is designed to detect and prevent. Systemic oversight functions as the care model, and EGCD names the syndrome that requires diagnosis and management [7]. The relationship to representational veracity is equally central. EGCD does not replace representational veracity; it depends on it. When descriptors, disease categories, population labels, provenance records, or community categories become inaccurate or ethically misleading, downstream governance is impaired even when data are de-identified and legally accessible, and representational failure becomes a governance dissolution problem once no authority can detect, adjudicate, or remedy it [18]. The relationship to the continuity trap is diagnostic. A dataset may appear continuous because it carries a stable identifier, provenance trail, data access agreement, or IRB number, while continuity in one artifact conceals discontinuity in consent scope, community standing, classification, deployment context, or algorithmic performance. The continuity trap names that proxy closure error, and EGCD describes the cumulative state that emerges when the error is repeated across the lifecycle [19].

### Limitations

This framework has several limitations. It is conceptual and requires empirical validation, and the score and staging thresholds are not validated metrics. The Royal Free–DeepMind Streams example is an illustrative application based on public records, not a full empirical case study or legal determination. The officer and review board functions may be difficult to implement unless adapted to existing governance structures. The framework does not resolve the political economy of DSHR, including commercial incentives, asymmetries between data contributors and data users, and global inequities in bargaining power. Community governance is not always straightforward, because communities may be multiple, overlapping, contested, or difficult to identify after aggregation. Future research should validate the scoring system across retrospective and prospective cases; examine whether the CAM improves IRB, REC, and DAC deliberation; test implementation in biobanks, cancer registries, health data platforms, and AI partnerships; compare community perceptions of dissolution risk; and evaluate whether adoption of the ECGRM reduces governance failures, improves trust, and preserves scientific value. Interrater reliability testing and prospective implementation research should precede any use of the thresholds for institutional performance assessment.

### Conclusion

EGCD names a central failure mode in DSHR: the loss of governance authority across the lifecycle of data, models, and biological materials. It is not enough to ask whether data were once consented, reviewed, de-identified, documented, or validated. The operative question is whether current use remains authorized, reviewable, classifiable, representationally faithful, algorithmically assured (where relevant), and remediable. EGCD provides a way to diagnose when those authorities have dissolved and to manage the problem through lifecycle governance. The task for global DSHR governance is not to preserve every original condition forever but to ensure that governance authority does not silently disappear while data, models, and biological materials continue to affect the lives, identities, and community standing of the persons and groups from whom they were derived.

## Data Availability

No primary data were generated or analyzed in this study. All cited sources are publicly available.

## Abbreviations

AI: artificial intelligence
CAM: Continuity Authority Matrix
DAC: data access committee
DSHR: data science health research
ECGRM: Ethical Continuity Governance and Response Mechanism
EGCD: ethical governance continuity dissolution
IRB: institutional review board
NHS: National Health Service
REC: research ethics committee
